# Preconception paternal bisphenol A exposure induces sex-specific anxiety and depression behaviors in adult rats

**DOI:** 10.1371/journal.pone.0192434

**Published:** 2018-02-08

**Authors:** Ying Fan, Chong Tian, Qilin Liu, Xianyue Zhen, Hui Zhang, Liangneng Zhou, Taibiao Li, Yun Zhang, Shibin Ding, Dongliang He, Xin Jin, Jian Liu, Beibei Zhang, Nannan Wu, Anne Manyande, Maoshu Zhu

**Affiliations:** 1 Department of Psychiatry, The Fifth Hospital of Xiamen, Xiamen, PR, China; 2 School of Nursing, Tongji Medical College, Huazhong University of Science and Technology, Wuhan, PR, China; 3 Department of Gastroenterology, The Fifth Hospital of Xiamen, Xiamen, PR, China; 4 Department of 110, Wuhan Mental Health Center, Wuhan, China; 5 Department of Neurology, The Fifth Hospital of Xiamen, Xiamen, PR, China; 6 Department of Respiratory, The Fifth Hospital of Xiamen, Xiamen, PR, China; 7 Department of Chinese Medicine, The Fifth Hospital of Xiamen, Xiamen, PR, China; 8 Department of Nutrition and Food Hygiene, School of Public Health, Xinxiang Medical University, Xinxiang, PR, China; 9 Department of Nutrition and Food Hygiene, School of Public Health, Tongji Medical College, Huazhong University of Science and Technology, Wuhan, China; 10 School of Psychology, Social Work and Human Sciences, University of West London, London, United Kingdom; 11 Central laboratory, The Fifth Hospital of Xiamen, Xiamen, PR, China; Radboud University Medical Centre, NETHERLANDS

## Abstract

Bisphenol A (BPA), an environmental endocrine-disrupting compound, has drawn a great attention for its adverse effect on behavioral development. Maternal exposure to this compound has been reported to induce anxiety and depression in offspring, but the effect of its paternal exposure is rarely discussed. This study investigated whether preconception paternal BPA exposure can affect the emotions of male rats and their offspring. Eighteen adult male rats (F0) received either a vehicle or 50 μg/kg/day BPA diet for 21 weeks and were then mated with non-exposed females to produce offspring (F1). The affective behaviors of F0 and F1 rats were evaluated in the open-field test, the elevated-plus maze and the forced swimming test, and their serum corticosterone were then examined. BPA exposure induced increased anxiety behaviors along with increased serum corticosterone in F0 rats. This paternal exposure also led to increased anxiety behaviors in F1 females and aggravated depression behaviors in both sexes of F1 rats. Furthermore, only F1 females exhibited increased serum corticosterone. Overall, these data indicate that preconception paternal exposure to a low dose of BPA may induce transgenerational sex-specific impairments in the affection of adult rats.

## Introduction

Bisphenol A (BPA), member of a class of endocrine disrupting chemicals (EDCs), has been widely used in food containers and packages for many years, and human beings are widely exposed to BPA[1]. Oral exposure is thought to be the primary route of BPA exposure in the general population[2]; for example, BPA was detected at nanogram levels in food in China in our previous study (e.g., the mean value was 7.708 ng/g fresh weight in cereals and cereal products)[3].

As a kind of xenoestrogen, BPA exhibits a very high affinity for estrogen-related receptor γ[4], which is expressed at higher levels in fetal brains than estrogen receptor α or estrogen receptor β[5, 6]. Moreover, BPA can cross the blood-brain barrier, cross the placenta, and enter breast milk[7]; thus, the effect of BPA on human brain and behavior interests many researchers. Prenatal and early-life stage exposure to BPA have both been reported to induce adverse behavioral problems including hyperactivity, inattention, conduct problems and increased levels of anxiety and depression in children[8]. In addition, evidence indicating BPA exerts adverse effects at low doses is mounting. A dose of 50 μg/kg/day has been thought to be a safe dose according to the current USEPA's reference(http://www.epa.gov/iris/subst/0356.htm). BPA exposure at low levels (below 50 μg/kg/day) has also been reported to induce behavioral changes[9–12], cognitive deficits[13–15]and increased anxiety behavior[15]. Special concern has been raised regarding BPA exposure in pregnant women and children; BPA is banned in food packing and containers for babies in many countries, and pregnant women are reminded to be cautious when using plastics.

Compared with maternal and childhood exposure, the effect of paternal exposure to BPA is less discussed. However, evidence shows that paternal exposure to BPA could affect the offspring generation and is associated with decreased birth size, increased gestational age[16], reduced sperm quality[17] and spatial memory impairments[18]. However, whether paternal exposure to a low dose of BPA can affect behavior remains unclear. Here, we investigated the affective levels in F1 offspring to assess the consequences of paternal BPA exposure at a dose of 50 μg/kg/day. We examined the anxiety behaviors by the open-field test and the elevated-plus maze, while assessed the depression behaviors by the forced swimming test. Finally, we analyzed the serum corticosterone, a key marker of hypothalamic–pituitary–adrenal (HPA) axis [19, 20], before and after the final behavioral test in order to gain further insight into the underlying mechanisms.

## Materials & methods

### Animals

Eighteen 60-day-old Wistar male rats (F0) were obtained from Hubei Research Center of Laboratory Animals, China. Animals were maintained in accordance with the Guidelines for the Care and Use of Laboratory Animals (Institute of Laboratory Animal Resources, National Academies Press, Washington DC, 1996). The animals were housed individually in polypropylene cages in special pathogen-free conditions in an environmentally controlled room (temperature, 21 ± 1°C; relative humidity, 60 ± 10%). Glass water bottles were used and a 12-h light/dark cycle (lights off from 18:00 to 06:00) and ad libitum access to food and water were maintained. F0 rats were randomly assigned to receive either a control or a BPA-containing diet every day for 21 weeks. Then, each male rat was mated to two unexposed female Wistar rats. The breeding procedure was repeated 3 times. Successful mating was determined when a vaginal plug was observed. The day of delivery was recorded as postnatal day (PND) 0. The litter size of each dam was randomly standardized to 8 pups(four/sex/litter) on PND 5. Pups were weaned on postnatal day (PND) 25 and then housed in pairs. BPA treatment had no effect on body weight, litter size or sex ratios for all rats (data not shown). Ethical approval was granted by the Ethics Committee of Tongji Medical College, Huazhong University of Science and Technology, Wuhan, China.

### Diet and BPA treatment

A semi-purified, phytoestrogen-free powdered formula (AIN-93G, Test Diet) was purchased from Beijing HFK Bio-Technology Co., Ltd., China, and used as the basal diet. Corn oil was dissolved with BPA from Sigma-Aldrich (CAS no. 80-05-7, purity 99%) to make a BPA stock solution. Description as our previous study[18], an automated algorithm calculated the necessary volume of stock solution based on the daily body weight of each rat. This solution was diluted in corn oil to form a total volume of 0.5 ml, which was then directly added into a 5g basal powder diet and mixed. Finally, the mixture was placed into a robust ceramic jar in the home cage of each BPA rat. The control group received a basal diet mixed with the same volume of corn oil as the BPA group. Basal chow was supplied after the rats ate 5g of chow.

### Behavioral tests

The F0 rats underwent an open-field test at two weeks after mating. The F1 rats underwent an open-field test, an elevated-plus maze and a forced swimming test on PND 56. The apparatus was cleaned after each test. Water was changed after every 4^th^ rat in the forced swimming test. All data were recorded with a computer-based tracking system (EthoVision®, NOLDUS). All tests were conducted between 9:00 and 10:30 to minimize circadian effects.

#### The open-field test (OFT)

Each rat was placed at the same spot in the square field (80 × 56 × 40 cm) apparatus at the beginning of the test. Each rat could move freely in the apparatus for 5 min. The percentage of time spent in the center zone was evaluated as an index of anxiety, and the moving distance was recorded as an index of locomotor activity.

#### The elevated-plus maze (EPM)

The elevated-plus maze consisted of a 10 × 10 cm center area with 2 open arms (50 × 10 cm) and 2 closed arms (50 × 10 cm). Each rat was placed in the center square facing a closed arm at the beginning of the test and then allowed to move freely for 5 min. The duration and entry times in the open arms were recorded as negatively correlated indicators of anxiety behavior, and the total moving distance was evaluated as an index of locomotor activity.

#### The forced swimming test (FST)

Each rat was put into a cylindrical water tank (50 cm high, 30 cm in diameter) filled with 30 cm of tap water (24 ± 1°C). To help rats adjust to the water, we allowed the rats 5 min in the water and then dried them and put them back into their cages on the first day. On the second day, the rats spent another 5 min in the water, and the latency to immobility, immobility and resting episodes were recorded as indexes of depression behaviors.

#### Serum corticosterone

To detect the changes of serum corticosterone level, tail blood was collected one day before and about 30 min after the final behavioral tests. The blood samples were centrifuged in an Eppendorf centrifuge at 4000rpm for 10minutes. Serum was aliquoted and frozen at −80°C until analysis. Each serum sample was analyzed in duplicate with ELISA kits (Nanjing Jiancheng Bioengineering Institute), following the manufacturer’s protocols.

### Statistics

All data were analyzed using GraphPad Prism (GraphPad Software) and represented as the mean ± the S.E.M. After confirming that data were normally distributed using the Kolmogorov–Smirnov test, comparisons between two groups were made using *t*-tests in F0 rats. The two-way ANOVA with post-hoc LSD tests were used for F1 rat data. Statistical differences were considered significant when the *P* value was below 0.05.

## Results

### BPA increased the anxiety behaviors of F0 rats in an OFT

**Fig 1:**
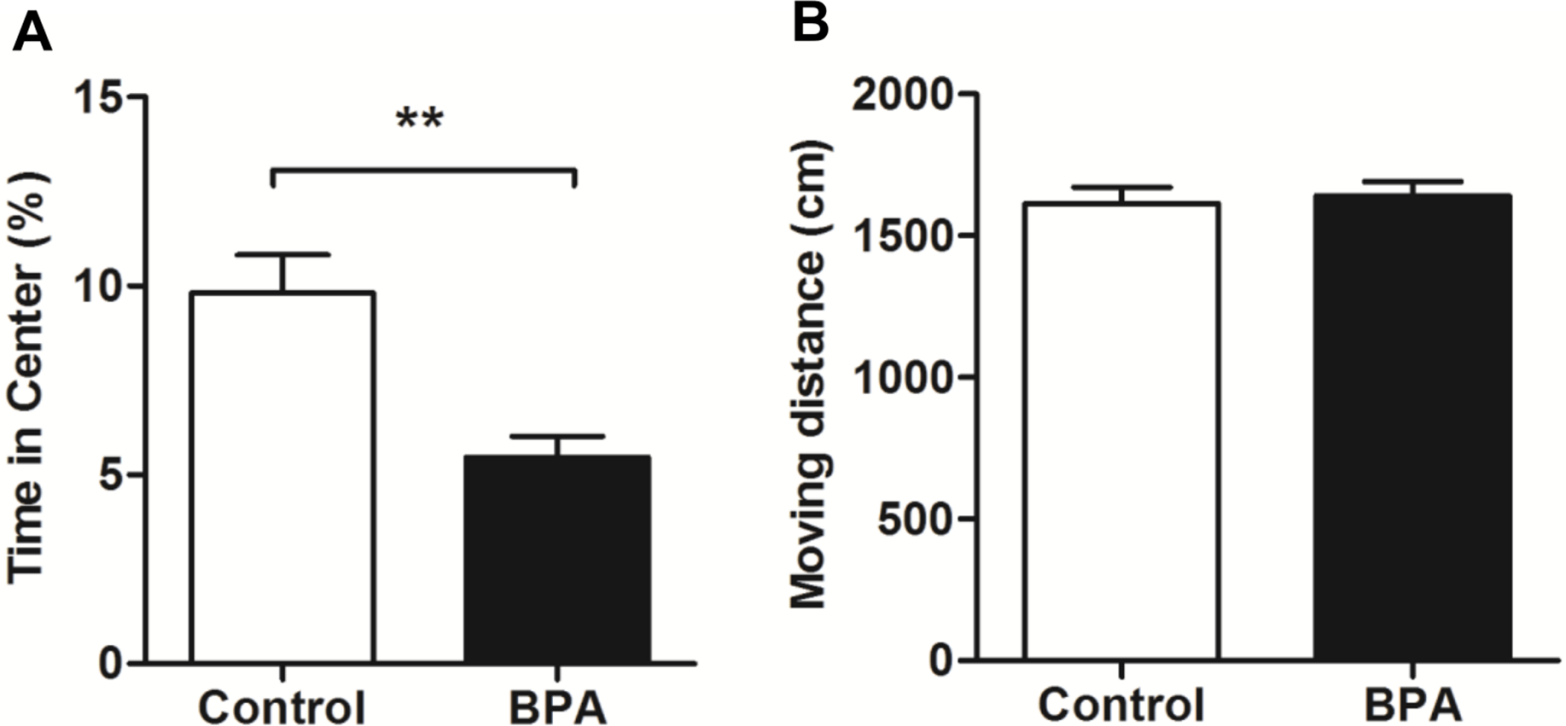
Effects of BPA exposure in F0 rats in an OFT. The OFT was performed by adult male rats who had received either a vehicle or a 50 μg/kg/day BPA diet for 23 weeks. (A) BPA-treated rats showed less time in the center of the box in an open field. (B) There were no significant differences in moving distance between BPA and control group. Mean ± S.E.M., *n* = 9, *P < 0.05, **P < 0.01, F0 control vs. F0 BPA.

To investigate the effect of BPA on anxiety behaviors of F0 rats, we evaluated their performances in an OFT after 23 weeks of BPA exposure (*n* = 9, each). The results showed that BPA rats spent less time in the central zone than the control animals (*P* < 0.01). No difference was observed in the moving distance between the two groups (Fig 1). These data suggest that BPA induces increased anxiety behaviors but not a locomotor-derived change.

### BPA induced aggravated changes in serum corticosterone in F0 rats after an OFT

**Fig 2:**
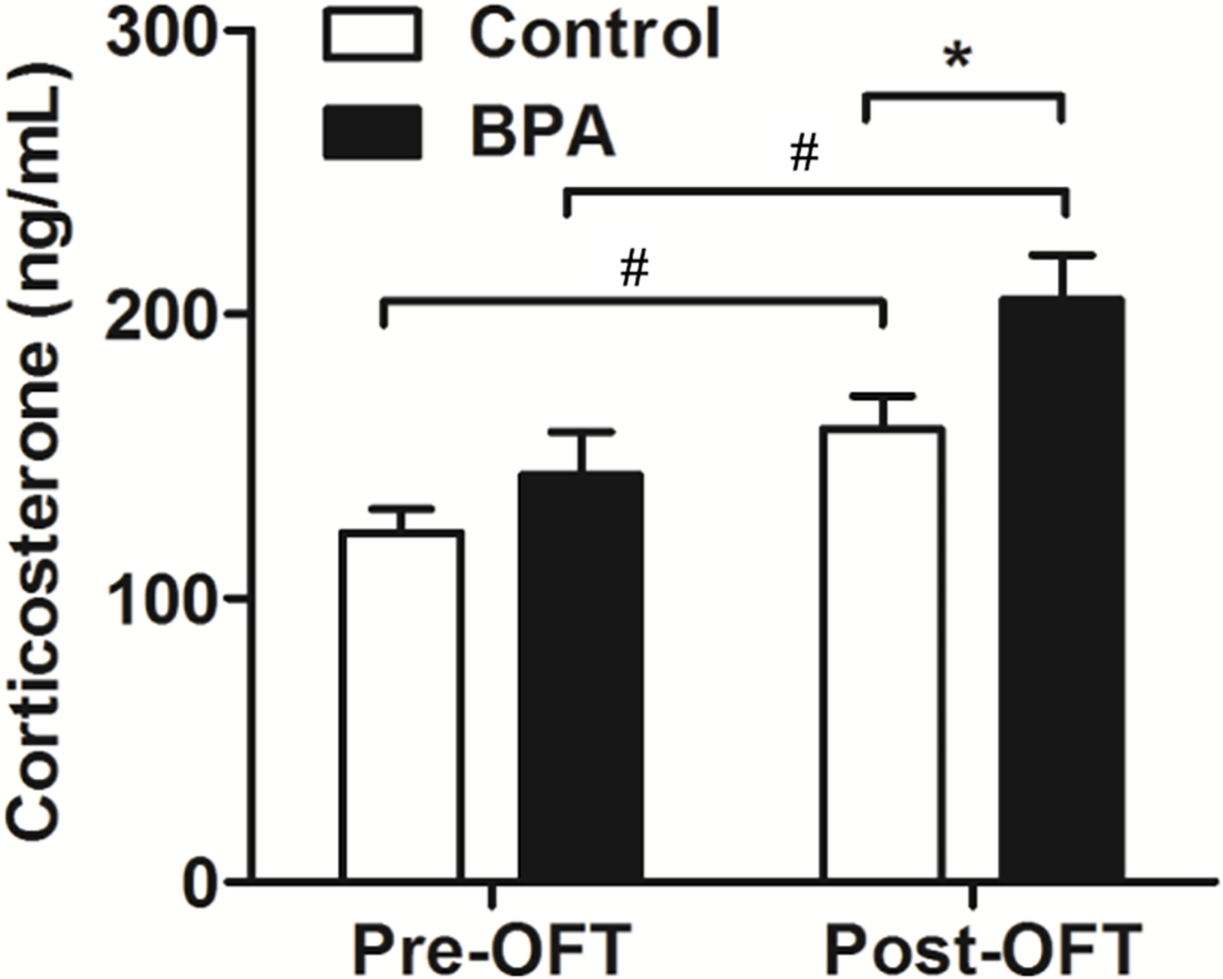
Effects of BPA exposure in F0 rats in the serum corticosterone level. The serum corticosterone level was detected by Elisa kit one day before and about 30 min after OFT. All rats showed increased level of serum corticosterone after OFT. F0 BPA rats showed increased level of serum corticosterone after OFT as compared with the controls. Mean ± S.E.M., *n* = 9, ^#^ *P* < 0.05 pre-test vs. post-test; * *P* < 0.05, **P < 0.01, F0 control vs. F0 BPA.

To determine if BPA might change the serum corticosterone, we examined the tail blood sample from F0 rats one day before and about 30 min after OFT (*n* = 9, each). The serum corticosterone showed no difference for the baseline level, but increased significantly after OFT (*P* < 0.05) in both groups. Meanwhile, BPA rats had significantly higher serum corticosterone after OFT as compared with the controls (*P* < 0.05) (Fig 2).

### Paternal BPA exposure induced sex-specific anxiety behaviors in F1 rats in an OFT

**Fig 3:**
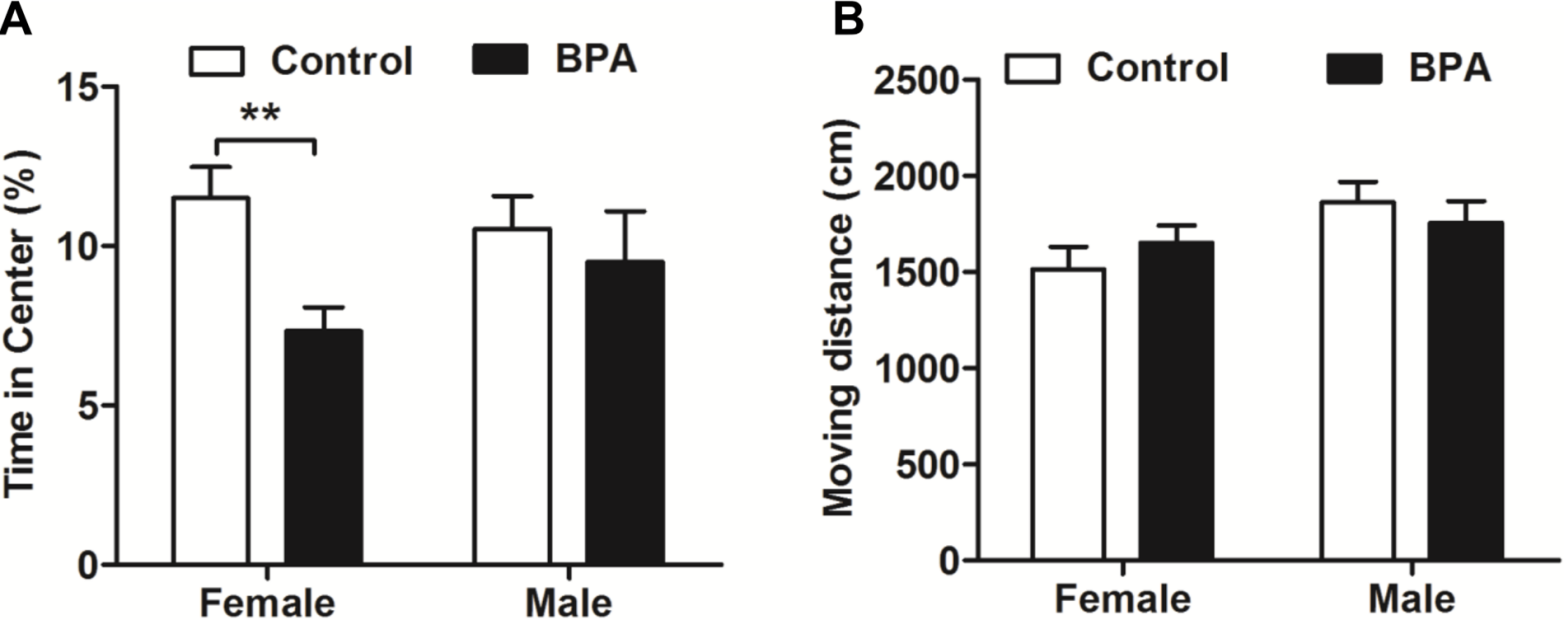
Effects of paternal BPA exposure on F1 rats in an OFT. The OFT was performed in F1 rats on PND 56. (A) F1 BPA females spent less time in the center of OFT than did the same-sex controls. (B) There was no difference in moving distance for all groups. Mean ± S.E.M., *n* = 9, *P< 0.05, **P< 0.01, F1 control vs. F1 BPA.

The anxiety levels of F1 BPA rats were also evaluated in an OFT on PND 56 (*n* = 9, each). F1 BPA females but not males spent less time in the center zone than did the same-sex controls (*P*<0.01). No difference was observed in moving distance between same-sex groups. In addition, there were no sex differences in any parameter within each group (Fig 3). These data suggest that paternal BPA exposure can increase anxiety behaviors in females but not in males.

### Paternal BPA exposure induced sex-specific anxiety behaviors in F1 rats in an EPM

**Fig 4:**
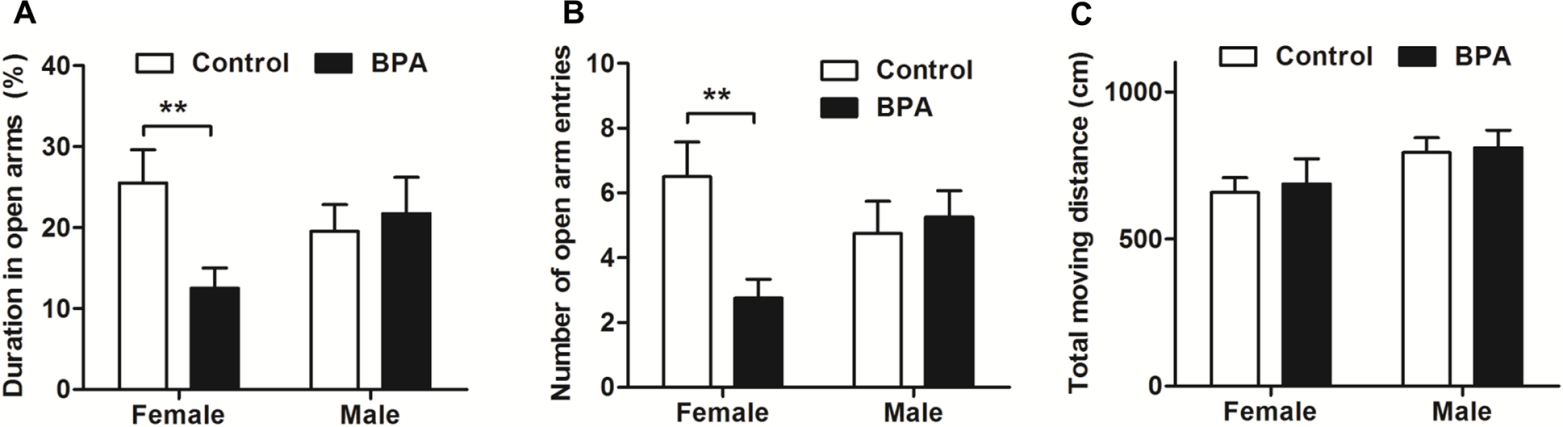
Effects of paternal BPA exposure on F1 rats in an EPM. The EPM was performed in F1 rats on PND 57. F1 BPA females spent less time staying (A) and entering (B) in the open arms of the maze than did the same-sex controls. (C) There was no difference in moving distance for all the groups. Mean ± S.E.M., *n* = 9, * *P* < 0.05, **P < 0.01, F1 control vs. F1 BPA.

We also estimated anxiety behaviors in F1 rats by EPM (*n* = 9, each). Consistent with the above results, F1 BPA females but not males showed less time spent in (*P* < 0.01) and entering (*P* < 0.01) the open arms compared with the same-sex controls. There was no difference between different sexes within the groups for any parameter (Fig 4). These data indicate that paternal BPA exposure may induce higher anxiety level in females but not in males.

### Paternal BPA exposure increased depression behaviors in both sexes of F1 rats in an FST

**Fig 5:**
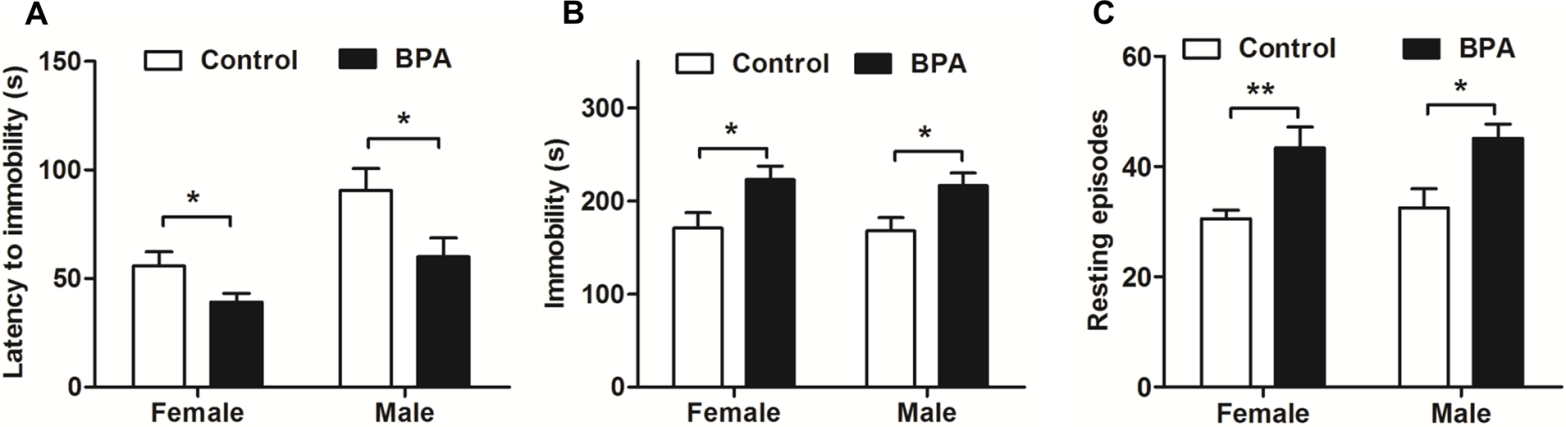
Effects of paternal BPA exposure on F1 rats in an FST. The FST was performed in F1 rats on PND 59. Both sexes of F1 BPA rats showed lower latency to immobility (A) but more time in immobility (B) and resting episodes (C) than the same-sex controls. Mean ± S.E.M., *n* = 9, *P < 0.05, **P < 0.01 F1 control vs. F1 BPA.

As shown in Fig 5 (*n* = 9, each), both sexes of F1 BPA rats showed decreased latency to immobility (*P* < 0.05) and increased immobility (*P* < 0.05) and resting episodes (*P*<0.05) during the FST compared with same sex controls. There were no sex differences within each group for any parameter. These data suggest that paternal BPA exposure may induce higher depression behaviors in both sexes of F1 rats.

### Paternal BPA exposure induced sex-specific changes in serum corticosterone in F1 rats after behavioral tests

**Fig 6:**
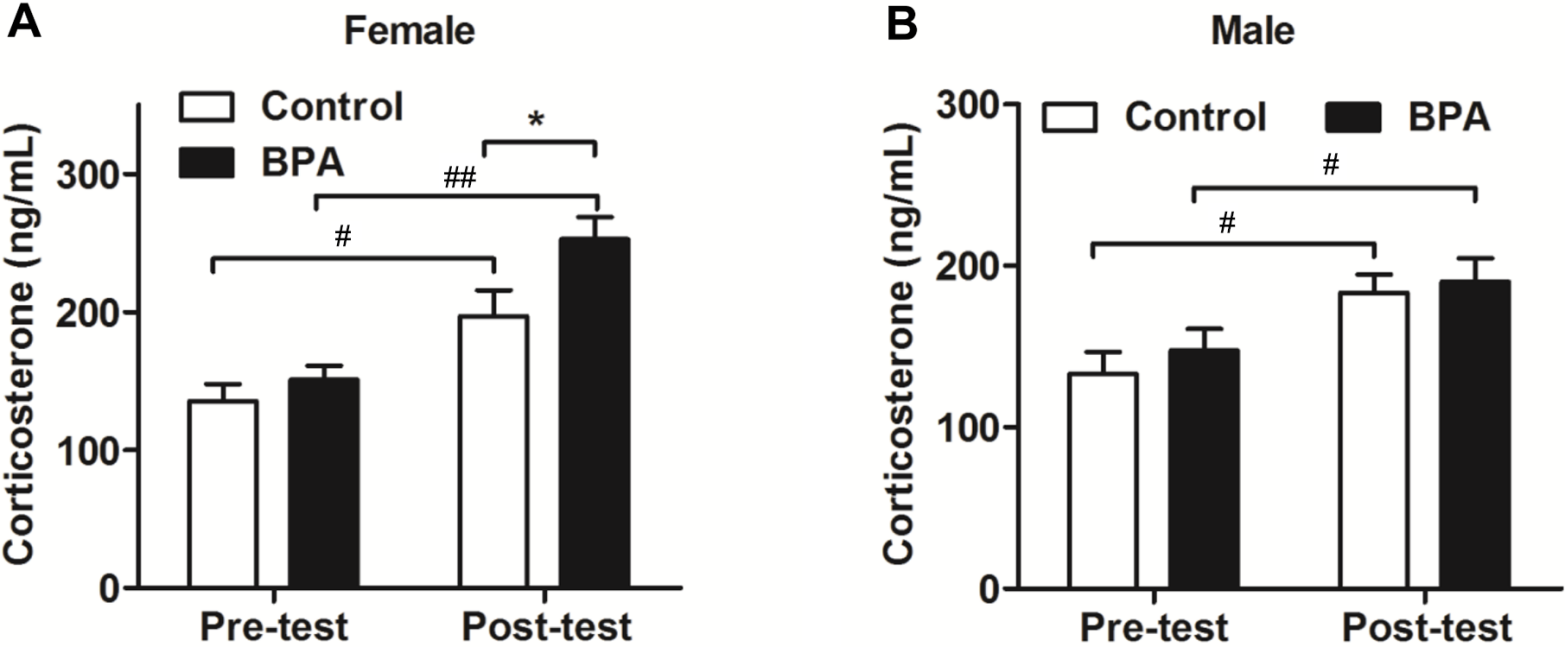
Effects of paternal BPA exposure on the serum corticosterone level in F1 rats. The serum corticosterone level was detected by Elisa kit one day before and about 30 min after the final FST. (A)The serum corticosterone was increased after behavior tests in two groups, F1 BPA females had higher serum corticosterone than the same-sex controls after behavior tests; (B) The serum corticosterone was increased after behavior tests within the groups, there was no difference at the point of post-test between the groups. Mean ± S.E.M., *n* = 9, ^#^ *P*< 0.05, ^##^*P*< 0.01 pre-test vs. post-test; * *P*< 0.05, F1 control vs. F1 BPA.

We tested the serum corticosterone one day before and about 30 min after the final FST (*n* = 9, each). As shown in Fig 6, the serum corticosterone was increased significantly after FST (*P* < 0.05) in both sexes of F1 rats. However, after the final FST, F1 BPA females showed significantly higher serum corticosterone than the same-sex controls (*P* < 0.01) (Fig 6A), whereas, the males had no difference between two groups (Fig 6B).

## Discussion

In the current study, exposure to BPA at 50μg/kg/day in adult male rats: (a) increased anxiety level, (b) aggravated changes in serum corticosterone, (c) transmitted higher anxiety and depression behaviors to F1 offspring in a sex-specific manner, (d) increased serum corticosterone only in F1 females. As far as we know, this is the first study to investigate the effect of paternal BPA on affective behaviors in rats.

In this study, the increased anxiety behaviors were expected based on results that F0 BPA rats spent less time in the central zone without locomotor change. Meanwhile, F0 BPA rats had higher serum corticosterone than the controls after the OFT. Some studies have showed that BPA increase rats anxiety behavior[21, 22]. These effects were partly interpreted as due to alterations in the hypothalamic–pituitary–adrenal (HPA) axis, such as increased corticosterone, adrenaline, et al[21, 23]. In line with these results, our data suggest that BPA may increase anxiety behaviors by modulating HPA axis in adult rats.

Interestingly, our data shows that the changes of emotion reaction induced by BPA were transmitted to the offspring in a sex-specific manner. As shown in Fig 3 and Fig 4, increased anxiety behaviors were documented only in F1 BPA females but not in males. Differently, both sexes of F1 rats showed increased depression behaviors during swimming in FST (Fig 5). Furthermore, the serum corticosterone was only increased in F1 BPA females, but no in males. This result suggests that paternal BPA may increase the susceptibility to anxiety response only in females but impair depression reaction in both sexes, which is not entirely due to the HPA axis. Anxiety and depression are two different emotions although based on some crossover mechanisms. BPA has been shown to induce stress hyperactive behaviors and higher depression responses in EMP and FST, through the dopaminergic and NMDA systems[24–26].Therefore, in the present study, besides HPA axis it is also possible that F1 BPA rats had changes in the other neurochemical systems involved in the affective response. Similar to our results, previous reports have acknowledged sex-specific dimorphic results after maternal BPA exposure in human. There are cohort studies showing an association between maternal urine BPA levels and anxiety and depression in girls, while some researchers found an association between maternal BPA exposure and anxiety and depression only in boys[27–29]. In addition, different behavioral outcomes in males and females caused by prenatal BPA exposure have been documented in animal experiments[30]. For example, prenatal exposure to bisphenol A impairs sexual differentiation of exploratory behavior and increases depression behavior in rats[31, 32]. Our previous study also showed that paternal BPA exposure can induce worse spatial memory impairments and higher stress response only in F1 females[18]. Although the underlying mechanisms of the paternal effects differ from that of maternal lineage, similar sex-specific effects may have occurred as early as the zygotic stage by epigenetic changes. It is well known that BPA can upregulate cluster in prostate and single strand DNA breaks in spermatozoa in adult male rodents[33]. As a result, some effects on the F1 rats must have been programmed into the genes in their germ cells and lead to deficits in brain and behaviors[34, 35].However, more studies are needed to confirm this hypothesis.

In conclusion, our data suggest that preconception paternal exposure to a low dose of BPA could induce sex-specific changes in anxiety and depression behaviors in F1 rats, which maybe by modulating HPA axis. These results provide a new perspective in the study of BPA-related behavioral alterations.
